# Implementing continuity of midwife carer – just a friendly face? A realist evaluation

**DOI:** 10.1186/s12913-020-05159-9

**Published:** 2020-04-15

**Authors:** Rhona J. McInnes, Alix Aitken-Arbuckle, Suzanne Lake, Caroline Hollins Martin, Juliet MacArthur

**Affiliations:** 1 School of Nursing & Midwifery, Griffith University, Gold Coast Campus, Gold Coast, Queensland Australia; 2 School of Health, Edinburgh Napier University, Sighthill Campus, Edinburgh, Scotland; 3 Nursing, Midwifery & Allied Health Professions, NHS Education for Scotland, Westport, Edinburgh, Scotland; 4 NHS Lothian, Edinburgh, Scotland

**Keywords:** Midwifery, Continuity of carer, Organisational change, Service reconfiguration, Evidence based care

## Abstract

**Background:**

Good quality midwifery care saves the lives of women and babies. Continuity of midwife carer (CMC), a key component of good quality midwifery care, results in better clinical outcomes, higher care satisfaction and enhanced caregiver experience. However, CMC uptake has tended to be small scale or transient. We used realist evaluation in one Scottish health board to explore implementation of CMC as part of the Scottish Government 2017 maternity plan.

**Methods:**

Participatory research, quality improvement and iterative data collection methods were used to collect data from a range of sources including facilitated team meetings, local and national meetings, quality improvement and service evaluation surveys, audits, interviews and published literature. Data analysis developed context-mechanism-outcome configurations to explore and inform three initial programme theories, which were refined into an overarching theory of what works for whom and in what context.

**Results:**

Trusting relationships across all organisational levels are the context in which CMC works. However, building these relationships during implementation requires good leadership and effective change management to drive whole system change and foster trust across all practice and organisational boundaries. Trusting relationships between midwives and women were valued and triggered a commitment to provide high quality care; CMC team relationships supported improvements in ways of working and sustained practice, and relationships between midwives and providers in different care models either sustained or constrained implementation. Continuity enabled midwives to work to full skillset and across women’s care journey, which in turn changed their perspective of how they provided care and of women’s care needs. In addition to building positive relationships, visible and supportive leadership encourages engagement by ensuring midwives feel safe, valued and informed.

**Conclusion:**

Leadership that builds trusting relationships across all practice and organisational boundaries develops the context for successful implementation of CMC. These relationships then become the context that enables CMC to grow and flourish. Trusting relationships, working to full skill set and across women’s care journey trigger changes in midwifery practice. Implementing and sustaining CMC within NHS organisational settings requires significant reconfiguration of services at all levels, which requires effective leadership and cannot rely solely on ground-up change.

## Background

Good quality midwifery care saves the lives of women and babies, with continuity of midwife carer (CMC) a key component of this [1]. A growing body of evidence, including a Cochrane review of 17 randomised controlled trials of over 17,000 women [2] shows that, compared to fragmented models of care, CMC results in better, or at least as good, outcomes and greater maternal satisfaction [2–6]. Despite this evidence, fragmented maternity care remains dominant in many countries and efforts to implement CMC have tended to be small scale, slow to grow or not sustained [4].

In CMC women are allocated a named or primary midwife who provides all midwifery care through pregnancy, birth and the postnatal period. This differs from fragmented models where women receive care from different midwives depending on stage of childbearing, risk factors, their location and local services [2]. Around the world CMC has been implemented in different contexts with variation in: composition of the multidisciplinary team (MDT); degree of autonomous practice, and target populations, all of which will impact on implementation, outcomes and sustainability [2].

Various CMC models, such as Midwifery Group Practice (MGP), caseload, independent or team midwifery, offer different opportunities for a known midwife to provide care. For example, MGP or independent practice midwives provide all care, including being on-call for their own caseload births, whereas team midwives provide antenatal and most postnatal care for their own caseload but are on-call for all births in the team [2, 3]. Thus women in MGP or independent models are more likely to already know the midwife attending their birth. It is as yet unclear if this makes a difference to any outcomes. All continuity models have better outcomes than fragmented care models but there is no consistent evidence that one type of continuity model is better than another [2].

Our literature scoping identified several countries attempting to grow CMC as a nationally available model of midwifery care. In the last decade, Australia has introduced government policy, legislation and midwifery education standards to re-orientate maternity services to ensure more women have access to CMC [7]. Despite this, changes to mainstream service delivery and expansion of existing midwifery continuity models remains slow with reports of less than 10% [7] to 19% [8] of Australian women having access to CMC. New Zealand legalised midwives as autonomous health professionals and as Lead Maternity Carers (LMC) in 1990, enabling them to care for a caseload of women. Midwives can be hospital and/or community based, with most community-based midwives self-employed and contracting directly with the Ministry of Health (see [9]). Women choose who to engage as their LMC (midwife, GP or obstetrician), with most (over 80% in 2015) choosing a midwife [10]. In Denmark, where midwifery-led practice has been the standard for all women since midwives were ‘authorised’ 300 years ago, caseload midwifery has become increasingly popular with around 24% of childbearing women being offered it in some areas [11]. Midwives generally work in pairs, each having a caseload of around 60 women per year, and are on call for their or their partner’s caseload [11]. CMC is a key component of current United Kingdom (UK) maternity plans e.g. *The Best Start* in Scotland [12] and *Better Births* in England [13]. The importance of CMC has been acknowledged in the UK since the 1993 publication of *Changing Childbirth* [14], however implementation was often small scale or not sustained. Thus, despite our knowledge of CMC as a superior model of care, the known risks associated with fragmented care and on-going efforts to drive change, its uptake is slow and patchy. Understanding how CMC works might help to normalise it as a sustainable model of care.

This study uses realist evaluation in one Scottish health board to explore how CMC works, for whom, in what context and to what extent, and so inform sustainable on-going implementation and up-scaling within the context of *The Best Start* [12].

## Methods

Realist Evaluation (RE) is a theory driven approach which aims to understand how complex interventions, such as CMC, work. RE goes beyond answering if a programme works to answer how it works, for whom, to what extent and under what circumstances [15]. Programmes work by enabling or motivating individuals to change their reasoning or behaviour [16]. People’s responses vary according to circumstances, which is why interventions work in some contexts but work differently or not at all in others and explains why interventions cannot simply be transferred to another context and expected to achieve the same outcomes [16]. In RE ‘mechanisms’ are the responses which are triggered in various circumstances to produce specific outcomes [15] and understanding these is fundamental to RE [17]. The interaction between circumstances and mechanisms is frequently presented as Context + Mechanism = Outcome (CMO) [16]. RE recognises that the theory-based understanding of how a programme works can be replicated in different contexts [16], which is particularly useful for upscaling programmes, such as CMC, that have been shown to work in some contexts. RE’s sensitivity to context, which influences how an intervention unfolds, makes it appropriate for trying to establish what conditions and factors are pre-requisites for embedding CMC into routine care and ensuring positive outcomes [18].

### Setting - National Context

In Scotland, and other UK countries, NHS maternity services are provided free of charge and most midwives practise in the NHS and are bound by NHS ‘Agenda for Change’ terms and conditions [19] and government strategies or policies. A very small number of women are cared for privately by an independent midwife. Midwives are the first point of contact for all women, the lead carer for ‘low risk’ women and care coordinators for women with additional risk factors or complex needs, where the obstetrician would be the lead carer [20]. Antenatal (AN) care is usually provided in primary care or hospital settings or occasionally at home; women can birth their baby at home, in a freestanding or alongside midwife-led unit or in hospital; immediate postnatal (PN) care is initially in the birth location and then in the woman’s home for up to around 10 days. Care is frequently fragmented by location and care provider according to stage of pregnancy, medical or obstetric risk factors, maternal choice and the availability of facilities.

There are significant concerns about the UK midwifery workforce including high levels of stress, sickness and poor workplace cultures [21]. These factors, along with feeling unable to provide good quality care and disliking the current fragmented model, are driving midwives out of the profession [22, 23]. The 2018 *State of the Maternity Services Scotland* [24] report highlighted increasing midwifery vacancies (quadrupled in the previous 5 years); more vacancies remaining unfilled, and an increase in midwives leaving the profession. Although birth rates have fallen over the past 3 years more women are presenting with increasingly complex needs including pre-existing medical conditions, obesity and giving birth later in life (54% of babies in Scotland are born to women over 30 years).

### Local context

This study was set in one Scottish health board, which is served by two maternity hospitals and has around 9500 births per year. Previous attempts to introduce CMC in this area were small scale and not sustained. Our baseline survey [25], developed to understand the local context, highlighted that local midwives supported the principles of CMC but opposed its implementation [26]. We used the ecological model [27] to organise survey findings and examine factors at the micro, meso and macro levels, which was helpful for showing how influences at various organisational levels can impact on the clinical experiences of midwives and ongoing implementation and sustainability of CMC, Table 1.

**Table 1:** The local context for introduction of CMC*

Potential Facilitators	Potential Barriers
*Macro (organisation):*	
High level support for new model. Clinical outcomes relatively good but rising intervention and decreasing ‘normal’ birth drive the need to change. Women generally satisfied with care but want more opportunities to build relationships with midwives.	Shortage of resources, funds & community-based facilities. Current systems embedded in and developed for the fragmented model. Midwives leaving and fewer attracted in – leading to local and national shortages.
*Meso (practice context)*	
Experience varies by location but good AN continuity within some community teams, while others recognise a need to improve. High stress and poor work experience may facilitate change, i.e. midwives will want to work differently	Lack of consistent access to physical spaces for clinical practice. Increasing work load, poor work-life balance and high stress among midwives – may constrain change i.e. midwives feel too stressed or burnt out to consider change. Poor relationships within and between professional groups.
*Micro (midwife, relationship with women)*	
Some midwives welcome opportunities to build relationships and for holistic, woman centred practice. Some have had experiences of CMC. Limited opportunity for continuity & developing good relationships in current model but wanted by women and some midwives. Newly qualified midwives have positive experiences of CMC during undergraduate education.	Most midwives want to stay in fragmented model. Many express concerns about safety of new model (e.g. not having right skills in right place), its impact on personal life or that it will not be properly resourced. Most midwives comfortable with rostered shifts and managing their work within these distinct periods. Many believe relationships with women are already good, or that women get too much choice or expect too much. Many do not believe new model to be very different to current care or to offer better continuity

* Macro = actions & interactions at organisational & managerial levels and with midwives & MDT at other levels; Meso = actions and interactions within the CMC team, between CMC and non-CMC midwives and others in the MDT; Micro = actions and interactions between women and midwives and individual midwife attitudes & beliefs. Source: informal discussions, stakeholder interviews, baseline survey

### The Best Start continuity model

*The Best Start* plan [12] was informed by published evidence and national consultation with key stakeholders including women and midwives. Following publication, health boards could apply to be ‘early adopters’ to test implementation with some anticipating this would bring resources. Early adopter status was approved in September 2017 and despite no accepted definitions, evaluation tools or clear indication of additional resources an early, and ambitious, target proposed that CMC be achieved for 75% of women within 2 years.

*The Best Start* [12] advocates teams of 6–8 midwives each with Full Time Equivalent (FTE) caseloads of 35 women at any given time (i.e. around 40–42 per year). Women are allocated a ‘primary’ midwife who provides continuity, with support from the team and/or a ‘buddy’ midwife. Operationalising CMC has varied across the 14 Scottish health boards according to local facilities and concurrent care structures. In most health boards, midwives were encouraged to develop a way of working to suit their team while ensuring 24-h cover for team births through a combination of rostering and on-call. In our study area CMC started as a ‘test of change’ with a small team of midwives (6 FTEs), who volunteered to trial the model within the context of the dominant fragmented model [12].

### Data collection, sources & analysis

As clinical academics within the board, the researchers supported the design of service evaluation tools to monitor staff and women’s experience as part of the test of change. This involved regularly attending team meetings to facilitate reflective practice sessions to develop a force field analysis of the key factors affecting implementation and staff experience. Gibb’s reflective cycle [28] underpinned the process, which itself became highly valued by the CMC midwives, aligning to the perceived benefits of the refreshed model for clinical supervision [29] such as enhanced self-awareness, problem solving skills and resilience. In particular, the team acknowledged the benefit of reflecting together for promoting positive team dynamics. In addition to facilitating reflection, we were also able to provide information from local and national implementation meetings and signpost supporting resources, to assist the team with their implementation of the evolving CMC model; e.g. practice ideas and approaches being tested in other health boards. Each meeting was written up, approved by the CMC team and shared with local health board. We also attended local and national policy and implementation meetings and local working groups that aimed to operationalise the model of care. Data sources are summarised in Table 2.

**Table 2:** Data Sources

Source	Number	Dates	Type of data
**Sources informing initial programme theories**			
Published literature		From Nov 2016	Papers and reports
Stakeholder interviews	4 leaders at macro level	June – Sept 2017	Interview transcripts
Baseline survey	321 midwives responded	Oct – Dec 2017	Survey data including free text
Participant observations	Local and national events	From Jan 2017	Field notes
**Sources for testing programme theories (in addition to above)**			
Facilitated CMC Team Meetings	12 meetings	Dec 2018 - Aug 2019	Field notes & summaries
CMC team surveys	15 (Team 1: 11 surveys, 50 responses; Team 2: 2 surveys, 18 responses)	Jan -Sept 2019	Quality improvement survey data
Local implementation meetings		Feb 2017 - Sept 2019	Field notes
National implementation and leader meetings		May 2018 - Sept 2019	Field notes
Women receiving CMC from local teams	89 women	Feb – Nov 2019	Service evaluation questionnaire data including free text
Wider workforce not in CMC team	36 midwives: 12 community and 24 hospital based	Feb- March 2019	Audit data
Participant observations	National stakeholder events	March 2017 and May 2019	Field notes
One-to-one midwife interviews	9 midwives: 4 leaders (3 not in health board); 2 in CMC teams; 3 in standard model.	May – Aug 2019	Interview transcripts

### Recruitment of interview participants

Interview participants were purposively recruited to reflect their roles within the implementation process, Table 2. Participants were invited by email, word of mouth or flyer and provided written consent prior to taking part. A realist evaluation informed topic guide was developed to steer the interviews (Additional File 1).

**Fig. 1:**
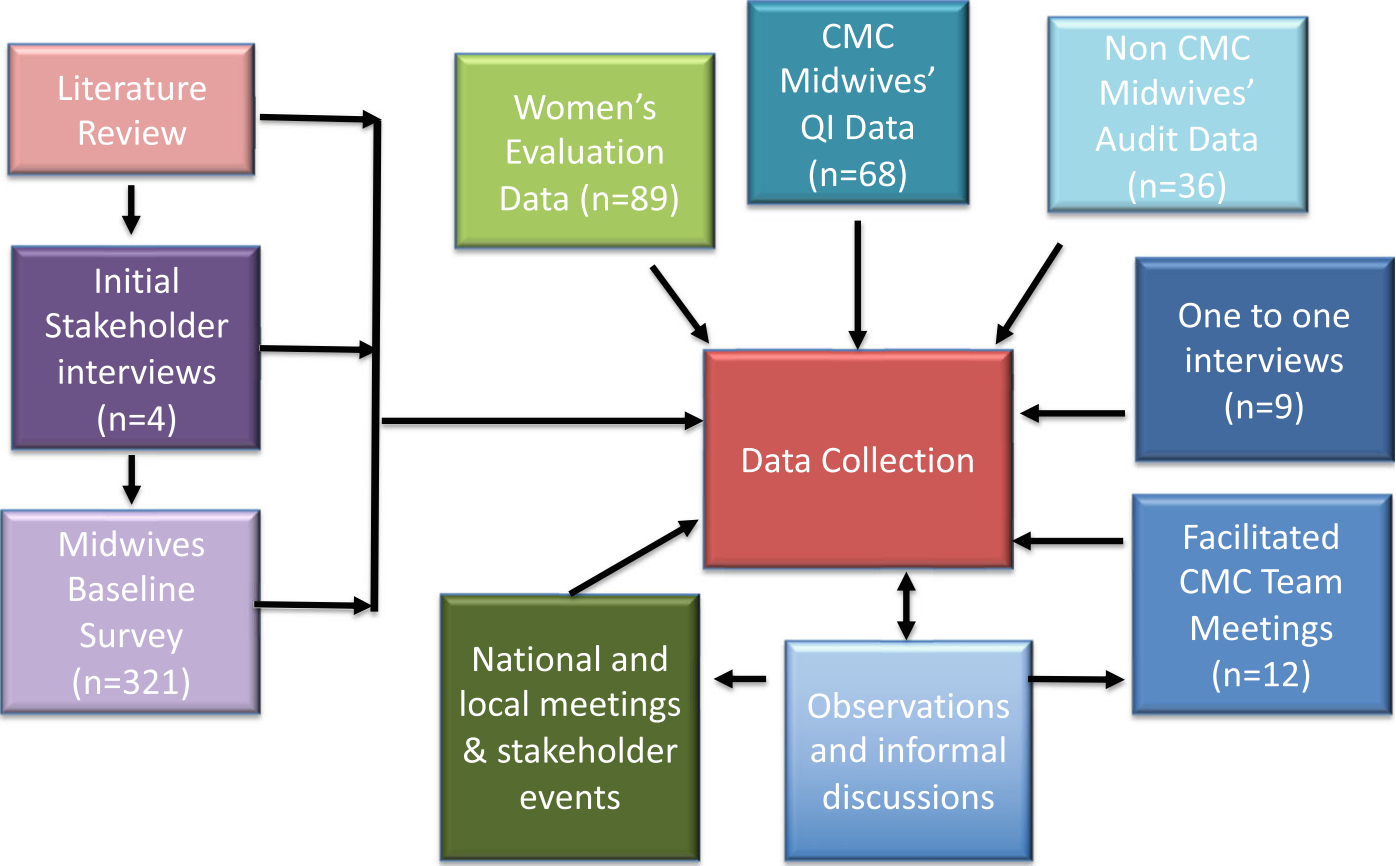
Data collection

The limited resource and time available to allow effective use of the developing evaluation process to inform implementation was challenging. Implementation was intended to be based on the Model for Improvement [30] but the iterative improvement cycles often became complicated by other changes, either at local or national level, as the CMC political landscape continually evolved. Implementation of the continuity model, collection of our evaluation data and analysis were therefore ‘messy’, iterative processes which frequently interacted as we went to and fro between a range of data sources, including experiential data, within a changing landscape, to identify, develop and test theories, Fig. 1.

### Analysis

RE begins with a programme theory, which is basically the premise within an intervention that ‘if we do X we bring about change Y’ [31]. Most programmes or interventions have a theory but do not always make this explicit. The first stage of our analysis was to identify candidate or initial programme theories. In our study these were informed by *The Best Start* document [12], the published literature, published and unpublished reports, local policies, listening to midwives’ responses to the publication of *The Best Start* [12] at formal and informal sessions and through interviews with policy and organisational level stakeholders. These theories were then tested and refined through data collection and analysis using the data sources outlined above, Table 2. Testing assesses if our expectations of how the programme works are true and, if not, enables us to explore why [32]. We developed 20 ‘if …then’ statements linking CMC to potential outcomes (Additional File 2) and, through discussions at team meetings, developed three CMO configurations (Additional File 3) as theories, which we tested during on-going implementation of CMC. ‘If-then’ statements form the initial steps linking programme elements with outcomes and have been used widely in realist studies [32]. Interviews were transcribed verbatim and uploaded into QSR NVivo (Version 12) along with meeting summaries, field notes, service evaluation and audit data. Data were coded according to our CMO configurations, either as part or whole CMOs. During testing and on-going implementation, we identified additional ‘if …then’ statements that explained some of the outcomes within our context, (Additional File 4).

### Ethics

We obtained ethical approval to conduct interviews and to use quality improvement and audit data in our research. We were advised that we did not need NHS Ethics review due to the nature of our data collection and participants. Ethical approval was therefore obtained from Edinburgh Napier University (SHSC 18014) with the main ethical issue being the risk of identifying participants from the CMC team, thus requiring careful management of confidentiality and protection of identity.

## Results

This section outlines the initial programme theory and then presents three inter-related programme theories (relationships, midwifery practice, and leadership and support) that were tested and drawn together into a refined programme theory that articulates the context, mechanisms and outcomes of successful CMC implementation. All data and quotes are identified by their source. To maintain anonymity midwives’ interviews are numbered 1–9 rather than by specific role or location, except where including role provides important context (and number is omitted).

### Initial Programme theory

The overarching theory is that CMC will improve clinical outcomes, increase care satisfaction and improve care giving experiences by changing the dominant model of care from a fragmented to a relationship-based model, which aligns with the published literature as discussed. The actual mechanisms by which CMC leads to better outcomes are not fully recognised, but where tested CMC reduces interventions and enhances normal physiological processes such as normal birth and breastfeeding. Causal mechanisms could potentially involve biopsychosocial interactions; for example, the complex hormonal interactions during pregnancy and birth are affected by socio-emotional factors, which are affected by relationships [33]. CMC provides opportunities for midwives and women to build relationships and get to know each other through repeated care contacts and for midwives, through being responsible for all midwifery care, to work autonomously to full scope of practice. *The Best Start* [12] also recommends aligning obstetric staff to midwifery caseloads to build professional relationships and collaborative working.

According to *The Best Start*’s theory several factors (relationships, full scope of practice, autonomy and collaborative working) might potentially act and interact to affect clinical outcomes and sustainability. In the literature, two key constructs dominated authors’ discussions of how CMC might result in better care experiences and outcomes and these aligned with our initial stakeholder interviews. These were relationships (midwife-woman; midwife-midwife, midwife-MDT and within the organisation) and how midwives practise (autonomous, flexible and to full scope of midwifery practice). In addition, stakeholders and some published papers highlighted the importance of good leadership and effective change management to enable CMC to be accepted and embedded in practice. The three programme theories are presented below, with the final CMO configurations in Table 3 and supporting evidence in Table 4.

**Table 3:** Refined CMO configurations

**CMO1: Good leadership builds trusting relationships which make staff feel safe, able to engage and practise autonomously thus supporting on-going implementation**		
CONTEXT	MECHANISM	OUTCOME
Leaders (organisation & policy) enact their vision & belief in CMC by: • Visible leadership • Congruence between vision and action • Appropriate resourcing of new model, including time, support • Trusting staff to be responsible / autonomous	Organisation Leaders (MACRO) • Direct resources to enable CMC • Challenge attitudes or behaviours that don’t support high quality care Midwives (MESO) • Feel safe, supported and reassured • Are motivated and empowered to engage • Trust the process of change will be well managed and properly resourced • Trust each other • Feel heard and understood	MACRO • Positive work place culture • Retention of staff • Sustained implementation of CMC MESO/MICRO • Role satisfaction • Wellbeing
**CMO2: In the context of effective leadership CMC provides opportunities to build more trusting relationships between midwives & women, at all levels of the organisation, which trigger changes in behaviours and practice.**		
CONTEXT	MECHANISM	PROXIMAL OUTCOMES
MICRO (woman-midwife) regular and repeated contact across the childbearing enable a relationship where: • Trust develops • Women don’t have to repeat their story so provide more information at care appointment • Midwives more informed and familiar with woman’s context • Women better informed about their care	Women • Feel known, understood & accepted rather than watched or judged, • Feel confident in their midwife’s abilities • Disclose more personal information & seek more information • Believe in/trust their midwife • Engage with health services and health advice • Feel empowered, confident in own abilities to birth & nurture their infant • Women & midwives feel relaxed and less anxious; Midwives • More informed about women’s circumstance, plan woman-centred, appropriate care, timely detection of changes requiring treatment or referral • Develop flexible communication/ keeping in touch so women feel safe and cared for	• Hormonal regulation optimises biopsychosocial processes • Health service engagement and healthy lifestyle choices • Woman centred care DISTAL OUTCOMES • Improved maternal & foetal wellbeing • Reduced clinical intervention • Increased breastfeeding • Increased satisfaction with care • Midwife role satisfaction and better emotional wellbeing
MESO: Prioritising space & time for team meetings. Collaborative working across organisation: • Builds trust • Shared philosophies & values • Open supportive communication • Shared understanding of roles	• Feel accepted rather than watched or judged • Feel confident, relaxed & less anxious about practice • Are able to ask for help or support, particularly in challenging situations • Communicate openly & honestly about personal & professional challenges (make CMC work for all midwives) • Value and care for each other • Supported and empowered to provide high quality care	• Midwife role satisfaction • Good work life balance • Reduced stress and anxiety • Safe high-quality care • Implementation of CMC
MACRO: shared meetings, genuine listening and addressing concerns: • Trust develops • Shared philosophies & values • Open supportive communication • Shared understanding of roles	• Feel valued, cared for, supported and empowered • Trusted to be in control of diary, workload and so able to provide flexible woman centred care • Motivated to engage with and support new model	• Safe high-quality care • Woman centred care • Autonomous practice • Implementation of CMC • Positive workplace culture • Retention of staff
**CMO3: in the context of good leadership, trusting relationships and autonomous practice midwives use the full scope of midwifery practice to provide flexible, woman-centred evidence-based care.**		
CONTEXT	MECHANISM	OUTCOME
Shared belief in the values and philosophies of CMC across organisation ensuring: • Relationship based care across the childbearing journey • Autonomous, responsible midwives practicing to full scope of midwifery • Choice of role / location • High quality evidence based care.	• Motivated to engage with CMC • Seek out best quality evidence to inform care • Reflect on consequences of care decisions & experiences to inform future care • In control of diaries, planning & flexible working • Provide home based care: empowering for women, build relationships • Provide expert, information & emotional support to women, midwives & MDT • Direct resources to enable CMC • Challenge attitudes or behaviours that don’t support high quality care	• High quality evidence-based care • Good clinical outcomes • Role satisfaction • Wellbeing • Positive work place culture • Retention of staff • Sustained implementation of CMC

**Table 4:** Evidence for cmo configurations

**1: Leadership and support** No I think it is down to leadership. Leadership at board level but then also the heads of midwifery giving leadership roles to appropriate people and giving them enough time to lead implementation; I think that’s really key as well … but that the senior leaders have vision and drive and are really wanting it to happen so they are helping motivate that devolved leadership I think (Midwife Interview 1) The senior managers who are I appreciate following government initiatives that are actually to implement this, but I think there’s a bit of a, I would say there’s a lack of faith. INT: What d’you think drives the lack of faith? RES: People don’t trust what’s going on. […] This feels very invisible, even though there’s lots of communication about it, it’s the nuts and bolts, people want to know how is this going to affect me? (Midwife Interview 3) I work in a board that’s very, very supportive of each other and any change, this is transformational change, but in any change we have a good I suppose executive nurse director and good lead and work as a team so therefore, and she’s very, very robust in the way that she carries any project through (Midwife Interview 4) It felt like they issued this report [Best Start] that this change is happening and that’s it and we’ve got to make it happen, and there was just very little information, had to sort of make it up as they went along. (Midwife Interview 5) Could there have been stages to the strategy, could there be a bit more instruction with the strategy about how this would be implemented? I know there were timelines but there wasn’t even a year to prepare, it was like ‘right, get on with it’. So I feel… so then the strategy has come out without people expecting it and then there wasn’t even time to talk to people about how they felt about it. (Midwife Interview 7) The team highlighted that having an approachable line manager ‘makes a difference’ to the transitioning to the new model. They highlighted how supported they felt and indicated that without this support and approachability they would have found the process significantly more challenging. (Summary of Facilitated CMC Team Meeting (FCTM) Jan 2019) There is a strong belief among the midwifery workforce that implementation of CoC will not happen. The resistance and concerns that were identified in the midwives baseline survey have not dissipated. The midwives are anxious and desperate for information about how a future model might work, were it to be implemented. (Summary of FCTM Jan 2019) **2: Trusting relationships** It was great to have continuity of care which made me feel safe and listened to (Women’s Evaluation) We had such a positive experience of pregnancy and birth, thanks to [the team]. Going into the labour room and being greeted by a familiar face was invaluable, and we felt so well cared for and supported. (Women’s Evaluation) Having the same midwife for me made the experience better than what it was like having my fist [sic] child. I built a great relationship with [name] and throughout my labour she knew exactly the way I wanted things done. (Women’s Evaluation) Had excellent midwifery care. There was a sense of investing in a relationship which made a positive difference to my birthing experience. I also felt confident as my midwife is experienced on labour ward (Women’s Evaluation) Team members are increasingly using each other for moral support. Each member is developing their own support network within the team (and for some outwith), and there is a strong sense of mutual respect and consideration for all members (Summary of FCTM Jan 2019) The team are really noticing the benefit of getting to know the women, and in their own environment. They report on the satisfaction this gives them in terms of being able to better relate to and understand the women’s preferences and needs, which helps them advocate better for the women, and the ‘nice’ feeling associated with developing a good working therapeutic relationship; the type of relationship they give high value to as part of good professional practice. (Summary of FCTM Feb 2019) I think what’s really important as well is that midwives need to be treated as respected professionals and adults, they also need to behave in that way but that often, that hasn’t been how services have been run and how midwives have experienced being a midwife, and so I think that means that we don’t get the best from them and how they could practice (Midwife Interview 1) So the midwife getting to know the woman and how she is and her behaviours and her background and her history so that it’s easier for the midwife to pick up any changes, I think that’s important, I think that would make a difference and I think also for the woman to have built up that relationship and trust already with a midwife and she feels comfortable with her, if there are any changes that she can disclose them, she feels comfortable to act, for example in labour, she feels more comfortable and relaxed because she’s got the midwife that she knows and trusts (Midwife Interview 5) You’re never going to be, like, one team with the people in the hospital cause they’re based in the hospital and that’s what they do all the time and they don’t, they’re not with you all the time, they don’t know what your work’s like on a day to day basis, you know, it is just being part of a separate team. And then they’re always going to have so many midwives that come in once it’s up and running, you know, I think it’d be quite difficult for them to get to know everyone and for it to feel like all one big happy family sort of thing. So I think it’ll always feel like you’re going into someone else’s workplace rather than… (Midwife Interview 9) **3: Practice change** They’re thinking about their practice and that’s [improving continuity] just an example of one of the things that they’re doing differently (Midwife Interview 4) So that [sickness in CMC team] provided opportunities for some midwives to go out and just cover for a short couple of weeks and one of the midwives didn’t want to come back, … actually none of the midwives wanted to come back and they’ve continued to work on that model, and because they’re coming into the hospital and working they’re talking to other midwives and we’re now getting quite a surge of midwives who want to work within the continuity models (Midwife Interview 4) Yeah it’s a different experience. You get to know the woman in her home and how she is in her own environment which I think is important, what her environment is and I think when they let you into their home then that’s again building up another layer of trust. (Midwife Interview 5) If you know the women better hopefully, I would imagine that means that then you have a much better understanding of that woman’s needs, her life, her home, her everything that then your care is more tailored to her and that presumably then has some impact on the outcomes. (Midwife Interview 7) And I think it’s [home visit] really important in changing the way that women view their pregnancies, … I think that by having all their checks with a midwife in a very clinical space that they feel the need to be in a clinical space when they need help, …, so I think early labour, … that women who want to see a midwife and they don’t feel comfortable being at home and they feel like they need to go into the hospital because they feel like they need something and they shouldn’t be at home. So …, I feel like our women have definitely been… I don’t think they’ve been going into the hospital as early and being sent home as often as the women I used to have and I feel like… Yeah, like, niggling or contracting regularly but they’re still quite mild, … so I don’t know if the women are feeling just more comfortable being in their own homes in labour and if they’re in labour during the day, like, we’re going out to pop in and be like ‘is everything okay, stay away from triage, don’t go in till you’re ready’ sort of thing. (Midwife Interview 9) Women seem more relaxed not being in a clinical environment and divulge more (Summary of FCTM Jan 2019) The significant majority of the team prefer the increased autonomy they have in relation to managing their work life. Being able to work from home, managing own diary and to an extent, working pattern, have been reported as positives and a few feel returning to shifts would now feel very constrained. However, others continue to find it difficult to get the balance (Summary of FCTM April 2019) The team are beginning to appreciate the enhanced job satisfaction working this model will be able to provide. They are enjoying the relationship the model enables them to build with the women and flexibility in the care they are able to provide (Summary of FCTM Jan 2019) One of the most significant themes … is the feeling of isolation within the labour ward; being in a room caring for a woman, not entirely sure of care management, equipment, or processes and not feeling able to access peer support if it is not proactively offered, possibly through fear of judgement or reprisal. This does not mean support is not available; rather it suggests a breakdown in communication between the midwives in the room and those they are able to access for advice or assistance (Summary of FCTM Feb 2019)	

Initially, due to their dominance in the published literature and in *The Best Start*, we assigned relationships to the first programme theory. However, following our testing in the implementation context we believe that ‘leadership and support’ is the most influential and it is in the context of effective leadership that trusting relationships and practice change are triggered.

### Programme theory 1: leadership and support

Organisational contextual factors can facilitate or constrain implementation [34–39] and these were reflected in our evaluation. Leadership, support from management and effective change management processes are hugely important during implementation [35, 39] and have also been shown to positively impact on midwives’ wellbeing [40]. Lack of good leadership with poor communication or failure to listen to concerns leaves midwives feeling undermined and unsupported. This leads to poor support for CMC across all levels of the organisation and restrains the development of trusting relationships. Pressure to make CMC work might reflect targets and timelines set by government, which combined with lack of resources, played out across all levels of the organisation including individual beliefs (micro)

I'm not averse to it once things have been ironed out, but people need to listen to the midwives and keep touching base with the midwives, and the impression I get is it's like 'well you haven't met this target, you haven't done this, you're supposed to be doing this', as opposed to 'why haven't you managed that, what's going on, what can we do to help, what can we make easier, how do we iron this out?' and that’s not the impression I'm getting from them, it's that 'you haven't done this, you haven't done that, you haven't done this either and why hasn’t that been done yet?' and that’s no way to work, it's no way to live. (Midwife Interview 6)

For CMC midwives in our study, organisational pressure to perform well [41] combined with micromanagement [42] stifled flexibility and undermined autonomous practice. As has been found elsewhere, lack of support and poor practice arrangements [43, 44] counteracted possible benefits and impacted negatively on midwives’ wellbeing and sustainability of CMC. In our study, as in others, there was (considerable) workforce resistance to change [36, 39, 42] much of which was predicated on concern about impact on personal lives and ultimately on wellbeing (Baseline Survey).

Good leadership of change management needed for CMC requires belief, shared vision and effective and consistent communication. During our evaluation, inconsistencies in beliefs at all levels affected how people engaged with CMC, e.g. whether to volunteer or to support implementation from within the wider workforce. Lack of belief in CMC or *The Best Start* was sometimes visible through inaction, reluctance to provide assistance or support, poor practice or incongruence between verbal and non-verbal communication.

### Refined theory 1: leadership

Good leadership builds trusting relationships which make staff feel safe, able to engage and practise autonomously thus supporting on-going implementation. Visible leadership provides shared visions and goals supporting midwives who might feel that CMC is a risk in a challenging practice environment, particularly if there is minimal system change. Good leadership may also buffer external pressures to implement CMC in unrealistic timescales. Poor workforce engagement and poor leadership make it difficult to sustain positive change at the level of the individual midwife. Lack of organisational alignment with genuine woman centred care and reluctance or fear of increasing autonomy in relation to ways of working does not support CMC implementation or sustainability.

### Programme theory 2: relationships

In the context of supportive organisational structures, CMC is founded on positive relationships between midwives and women [12, 38]. CMC aims to build relationships through frequent care contacts with the same midwife during a woman’s care journey [12]. These relationships result in better clinical outcomes and care experiences for women [36, 38, 41, 44–54] and are thought to work through various mechanisms such as: trust [38, 47–49, 52, 53, 55]; feeling known [49, 51, 52, 56]; feeling empowered or confident [52, 53, 57], feeling relaxed [52]; emotional support [45, 55, 58, 59]; advocacy [60] and feeling safe [47]. Women in our evaluation said they liked having a relationship with their midwife, ‘seeing a friendly face’, feeling known and feeling confident in their midwives’ abilities.

*‘One of the highlights was meet the midwife meetings. This left me and my partner very reassured and not scared about labour at all. My birth plan was known by the midwife delivering my baby. I think all of the above gave me the psychological comfort which helped me deliver my baby naturally and then breastfeed my baby from the very first hour of his life’* (Women’s Evaluation)

‘Their’ midwife not being present for their birth was disappointing for some but many accepted the early ‘testing’ stage of implementation. Perhaps of significant interest was how woman-midwife relationships impacted on midwives’ practice by sustaining them and motivating them to provide high quality, individualised, women-centred and evidence-based care. The responsibility midwives felt for their women encouraged them to reflect on and change their approaches (See Theory 3). However, the responsibility could also increase their feelings of anxiety if women did not have a good outcome or care experience, even if this was beyond their control.

Returning to the ecological model, relationships can also be understood to be working at micro (midwife-woman), meso (midwife-midwife and MDT) and macro (organisational) levels to influence staff wellbeing [36, 43, 44, 48, 61–63], optimal care practices [64] and CMC’s implementation and sustainability [43]. In our study CMC midwives were sustained and enabled by the relationships they developed within the team. These were particularly important at the outset when they were developing new ways of working. For midwives more used to working in the hospital context the team provided a sense of belonging. By contrast, relationships in the hospital or at the interfaces between the different models of care were more challenging. Again, positive relationships were sustaining for CMC midwives supporting their learning and ensuring they felt able to ask for help if required. Conversely, CMC midwives often felt watched and judged, which was undermining and disempowering. Feeling judged and perceiving a burden of high expectations from the maternity workforce through being the ‘test team’ for CMC meant they felt constantly alert and under pressure to perform well. In this context CMC midwives found it hard to ask for help, or if they did support was not always forthcoming, raising concerns about safe practice.

Key within relationships is trust [38, 47–49, 52, 53, 55], which also works within the workforce. Trusting relationships at all levels are fundamental to sustaining on-going implementation of CMC, to midwife wellbeing and to women’s positive experiences. More commonly, CMC midwives experienced a lack of trust, which played out through persistent questioning of skills, motives and commitment leading them to feel the need to continually justify their decision to volunteer for the change work and to prove worthy and hard working. Trust within the organisation is further discussed in Theory 3.

### Refined theory 2: relationships

Within the context of effective leadership, CMC provides opportunities to develop trusting relationships between midwives and women and within small CMC teams. These relationships improve women’s emotional wellbeing, support engagement with services and trigger changes to how midwives’ practise (see Programme Theory 3). Negative relationships at any level disempower midwives and constrain the full implementation and sustainability of CMC. The effect of resistance to CMC on relationships and indirectly safety, should not be underestimated.

### Programme theory 3: midwifery practice

Continuity midwives work differently from midwives in fragmented care [41, 51, 63]. This difference encompasses working more autonomously [36, 51], with greater responsibility [51, 65]; flexibly [44, 48, 51, 55], feeling in control [42, 66]; providing high-quality care that is evidence-based [41], holistic [45] and individualised/woman-centred [44, 53, 56], and working with the full scope of midwifery practice [1, 39, 51, 55, 67]. This different way of practising results in greater job satisfaction and emotional wellbeing for midwives [41, 55]. For example; working in CMC both enables and requires autonomy [41, 55], and professional autonomy results in better job satisfaction and wellbeing [7, 9, 40, 61].

Our evaluation data was consistent with the evidence that working to full scope of practice is enabled by CMC but constrained within fragmented models. Within our study the CMC midwives identified working across women’s childbearing journey as important for providing woman-centred care and for increasing their midwifery role confidence, sense of empowerment [55] and job satisfaction [67]. However, we also found that autonomy, flexibility and scope of practice were affected by practice context, i.e. midwives need to be in control of their own workload to be able to practise flexibly and autonomously. This requires the organisation, more used to a transactional management style to trust in midwives’ ability to manage their own caseloads and working patterns effectively. However, this relinquishing of power proved challenging thus hampering completely flexible and autonomous practice (see Theory 1). This has been identified by others; for example, in organisations, such as the NHS, ‘midwifery practice may […] be restricted by the misuse of policies, protocols and contractual or employers’ obligations’ [68], making autonomous practice potentially more difficult.

Implicit in the literature is the theory that exposure to the whole childbearing journey and working to full scope of practice has the potential to change midwives through personal and professional development, accountability, responsibility, reflective practice and being in control of their own diary and workload. We found that the women-midwife relationship is key to this practice transformation (see Theory 2). Positive experience of CMC then motivates midwives and managers to engage with the opportunity to implement CMC either by volunteering or actively supporting implementation from within the wider workforce. In our study, and the literature, midwives chose to trial (or work in) CMC models, which could suggest that they might be intrinsically different to other midwives who did not put themselves forward; for example, they may have a stronger philosophical commitment to physiological pregnancy and birth or woman centred care or have different personal attributes [51, 52, 69]. In support of this we identified that many of the midwives who volunteered to test CMC were motivated by a lack of belief in or negative experience of the current fragmented model. However, interviews with midwives and our field work with the teams also suggests that CMC changes the midwife:

*‘being involved in intrapartum care has really opened up my… made me more rounded as a midwife, I've been more interested in the care journey that the women will have through the intrapartum period. Normally [in previous role as community midwife] I would've discussed that with them but I might not have gone into as much detail or discussed the importance of really thinking about certain things and I wasn’t as invested I suppose in the intrapartum, didn’t see it as… it was the extra thing that I just probably never quite got round to. Whereas now I would see that as a bit more important than I would've thought before.’ (CMC Midwife; Face to face interview)*

Several studies have identified differences between midwives working in different models of care but were conducted after exposure to CMC, e.g. [7, 9, 11, 55]. However, two very small prospective studies [36, 70] showed positive changes in attitudes to professional role and role satisfaction following exposure to caseload/MGP working.

Although our initial focus was on how practice changed within the team, our evaluation highlighted that system change was needed otherwise ground-up change was too constrained to be effective and sustained. Maternity systems designed for the fragmented model act against CMC, much like they did following *Changing Childbirth* [44] and proved challenging during our test of change. CMC midwives experienced this through lack of mutual understanding of roles, conflictual care pathways, unclear referral processes and difficulty working in a wider system that had remained unchanged. When these situations arose, midwives resistant to implementation of *The Best Start* [12] would hail them as good reason to stop the change rather than assist to resolve the issues, increasing the sense of division with the CMC midwives. This highlights the importance of securing a shared vision and commitment across the workforce, not just within the CMC teams.

### Refined theory 3: changing practice

Within the context of effective leadership, trusting relationships and autonomous practice CMC midwives use the full scope of midwifery practice to provide flexible, woman-centred evidence-based care. This approach is not intrinsic since working in CMC and reflecting on their new approach to care changes how midwives practise. In the context of limited organisational support and poor relationships, attempting to embed CMC within the unchanged fragmented model in an unchanged system is not sustainable and efforts become increasingly fraught as CMC practice diverges from fragmented care.

Good leadership of the change management required for CMC requires belief, shared vision and effective and consistent communication. During our evaluation, inconsistencies in beliefs at all levels affected how people engaged with CMC e.g. whether to volunteer or to support implementation from within the wider workforce. Lack of belief in CMC or *The Best Start* was sometimes visible through inaction, reluctance to provide assistance or support, poor practice or incongruence between verbal and non-verbal communication.

#### Refined programme theory

**Fig. 2:**
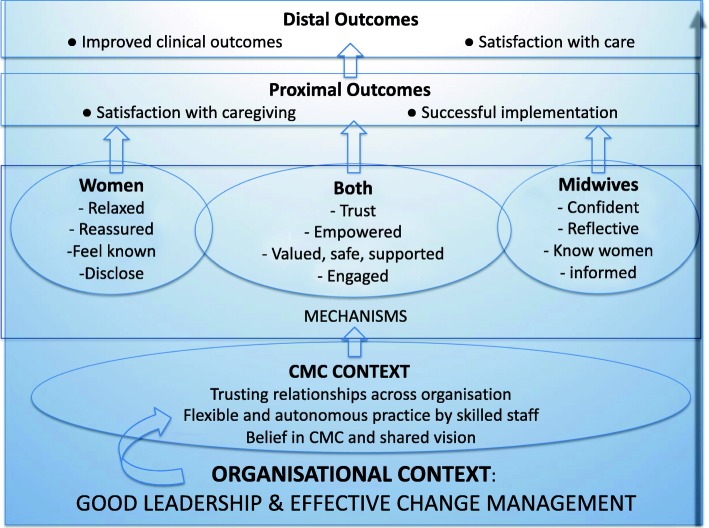
Refined programme theory

Good leadership and effective change management which prioritises the development of trusting relationships across all levels of the organisation enables CMC to be implemented. These relationships along with shared values and beliefs then become the context that actively supports midwives to be autonomous practitioners who feel empowered and confident in their practice and committed to provide genuine, flexible, woman centred/individualised care to their caseload of women. These in turn result in improved professional role satisfaction, wellbeing and retention within the workforce. Flexible, woman centred/ individualised care in this context enables midwives to get to know women, their care needs and their personal circumstances which allows them to plan care appropriately, tailor information to individual needs and, through early detection of change or risk, treat or refer early resulting in improved clinical outcomes and greater satisfaction with care. Women centred care situated within a trusting relationship optimises biopsychosocial processes by empowering women, improving their emotional wellbeing and enhancing their engagement with the health services, Fig. 2.

## Discussion

Effective leadership is essential for developing trusting relationships, the context in which CMC flourishes. Trusting relationships are the foundation on which CMC is built and become the cement that holds it together over time. Relationships matter, not only because women want them, but because it is the woman-midwife relationship that triggers change in women’s behaviour [69] and midwives’ practice resulting in better outcomes and care giving experience. The importance of woman–midwife relationships are already recognised, as are the supportive, sustaining and enabling relationships between midwives within CMC teams [48]. However, our study also suggests that good leadership operating across all levels is essential for building trusting relationships throughout the organisation, and that these relationships are required for midwives and others within the MDT to actively engage with CMC and change practice. Broken relationships (e.g. where managers do not trust midwives to act autonomously, where midwives do not trust managers to provide effective support or pressure to succeed) are barriers to practice change across all levels and to CMC implementation. This suggests that a shift from transactional to transformational leadership style within the organisation might help facilitate change.

Changes in practice, or personal and professional behaviours, of all professional groups (i.e. midwives and others within the MDT), across all levels and locations (i.e. CMC, wider workforce, organisational) are needed to accommodate CMC in the pre-existing fragmented model, while they co-exist. In this way CMC is a socially contingent complex intervention within a complex social system, i.e. its success relies on how people respond to CMC and how this affects their behaviour [71, 72]. Responses relate to personal beliefs or behaviours; those who believe in an intervention will enact their beliefs if the intervention provides resources or removes barriers [72]. All published studies identified, in keeping with this study, required midwives to volunteer to implement a new model of practice suggesting their reasoning already aligned with CMC and would implement change once any constraints were removed. Conversely midwives choosing to remain within the wider services may be less aligned with CMC indicating a need to change their reasoning or response to CMC as well as providing resources and opportunities [72]. Therefore, in a context where the operational implementation of CMC has been mainly rejected in advance [25] it is not enough to simply offer more opportunities to initiate or test more of the same. For example, rather than focusing only on behaviour, structural changes across the organisation and preparation of the future midwifery workforce might better support implementation and sustainability. We know that exposing student midwives to CMC during their undergraduate education increases their desire to work in CMC [73–75].

Allen et al., [69] identified ‘Synergistic Health Engagement’ as the mechanism by which optimal caseload midwifery (comprising philosophical commitment, institutional infrastructure and support, and personal attributes) modified the risks of preterm birth through women both attending and buying in (emotional investment and commitment) to services. In our study this mechanism might also explain the impact of caseloading on the midwife who through her relationship with women ‘bought in’ to CMC to ensure women had good experiences and outcomes.

Incorporating good evidence into practice is known to be slow [76] although in maternity care some evidence translates much quicker than others [77]. The inability to mainstream CMC, a high-quality evidence-based model supported by consumer demand, is particularly intransigent and [77] suggests that this might be because health professionals support change that aligns with their beliefs, biases and fears. Analysis of organisational context might also provide clues. Plamping [78] proposes that resistance to change in the NHS is due to four guiding principles which affect people’s behaviour: ‘can do should do’; ‘doing means treatment’; ‘treatment should fix’, and ‘I’m responsible’. She suggests that care is driven by treatment that fixes, which is why disease prevention and health promotion remain low priority despite our knowledge about societal determinants of ill health. Health professionals believing ‘I’m responsible’ will ‘struggle for dominance’ preventing cooperative or partnership working and excluding ‘patients’ from decision making [78]. The barriers to upscaling CMC become apparent when it is recognised that CMC requires autonomous midwives to work in partnership with women (first and foremost) and with other professional groups; to care or enable rather than ‘fix’, and to do less, but appropriately [79].

### Strengths and limitations

This evaluation was informed by a large amount of data from a range of different sources. However, our focus was mainly on one health board setting which has its own specific contextual factors and history and may, therefore, privilege some concepts over others or favour an area specific perspective. Our findings resonate with much of the published literature and benefit from real time exploration of CMC implementation, suggesting that as per RE the mechanisms uncovered may be transferrable.

### Recommendations for practice

- Good, visible leadership and effective change management strategies to change reasoning and to support those already bought in to CMC.
- Changes to organisational structures and practices across all levels, including relinquishing outdated controls that no longer serve a purpose.
- Trust in and empowerment of midwives as autonomous and responsible professionals.
- Ensure all models maximise relationships across the childbearing continuum. The possibility of a dose response should be considered and is worthy of further testing.
- Ensure exposure to the whole childbearing continuum and using full midwifery skillset as this changes practice.
- Facilitate sustaining relationships within the CMC teams.

## Conclusions

Trusting relationships are the foundation for CMC, an evidence-based model of high-quality midwifery care that has better clinical outcomes, greater satisfaction with care and better caregiving experience. Implementing and sustaining CMC within the wider organisational context of the NHS requires significant reconfiguration of services at all levels. This requires effective leadership and cannot rely solely on ground up change. Trusting relationships are key and require to be nurtured and sustained to bring about organisational and cultural change to ensure CMC becomes the norm.

## Supplementary information

**Additional file 1.** Interview topic guide.

**Additional file 2.** Initial ‘If… then’ statements (pre-testing): ‘If…then’ statements, developed from the literature, The Best Start plan and stakeholder interviews.

**Additional file 3.** Preliminary CMO configurations: these were developed from the ‘If…then’ statements and tested in the on-going evaluation.

**Additional file 4 **Additional **‘**If ……then’ statements from testing.

## Data Availability

The data used to inform this paper are presented within the paper or as supplementary files. Data sharing beyond this is not available as, other than interview transcripts, specific datasets were not generated by this study. Interview transcripts cannot be uploaded in full as this risks breaching confidentiality and because participants did not consent to full sharing of their transcripts.

## Abbreviations

AN: Antenatal
CMC: Continuity of midwife carer
CMO: Context mechanism outcome
FTE: Full time equivalent
LMC: Lead maternity carer
MDT: Multidisciplinary team
MGP: Midwifery group practice
NHS: National health service
PN: Postnatal
QI: Quality improvement
RCM: Royal college of midwives
RE: Realist evaluation
SG: Scottish government
UK: United Kingdom
