# Supplementary material for: Implementing continuity of midwife carer – just a friendly face? A realist evaluation
Source: BMC Health Serv Res. 2020 Apr 15;20:304. doi: 10.1186/s12913-020-05159-9 (PMC7158105; doi:10.1186/s12913-020-05159-9)
Supplement: Supplementary file 1 — Additional file 1. Interview topic guide. [file 12913_2020_5159_MOESM1_ESM.docx]

**New Topic Guides for Midwives in teams/ not in team/ MDT and other stakeholders**

**Introductory questions – for all**

1. Could you tell me what you think about CofC models?
2. What do you think would be an ideal CofC model?  What would it look like..?
3. Could you tell me about any difficulties you see with CofC models?

**Exploring context – for all**

1. What **staff characteristics** do you think help/**drive** CofC implementation, prompt: staff beliefs, the way they work, or are organised, experience?
2. What **characteristics/things** in your [team/area/Scotland] do you think are **slowing** **down** implementation of CofC? Eg resources, midwives, organisation, finances ...

**Exploring context and looking for mechanisms for successful CofC implementation**

1. There seem to be **external factors** affecting how CofC models are set up and how CofC progresses [across boards] … what do you think? Prompt: not staff.. go beyond staffing.
2. In your opinion how appropriate are the **resources** provided for this model?
3. How do you think the **level of experience of CofC** [in the team/health board/ MDT] influences how [CofC/the team] develops?
4. How do you think model impacts on how you/midwives/MDT provide you/their care? I’m thinking that you/they **may be doing things differently** than you/they used to do before the new model was implemented?
5. How do you think the new model affects the way midwives and the multi-disciplinary team works as a **team**?
6. There’s evidence that CofC leads to **better outcomes** for women, why do you think this is? What is it about CofC that makes the difference?
7. In your opinion is it important that **midwives volunteer or choose** to work in CofC models? Prompt: go beyond personal to explore why / how this might impact on implementation / outcomes

**Looking for known, unknown intended and unintended outcomes – various**

1. Tell me about **any changes** you see in your own professional practice because of the new model?
2. Are there any important things that are **working well** for your professional practice because of the new model?  (or that you see working well if not in CofC team)
3. I am just wondering whether the new programme may create **higher stress** levels than usual and how that has affected the relationships with colleagues?

**Other questions if time**

1. In your opinion what is **essential** to make CofC work ie for women to achieve continuity of carer?
2. How do you think is CofC can be **sustained** long term? I’m thinking resources, changes, people….?
