# Supplementary material for: Implementing continuity of midwife carer – just a friendly face? A realist evaluation
Source: BMC Health Serv Res. 2020 Apr 15;20:304. doi: 10.1186/s12913-020-05159-9 (PMC7158105; doi:10.1186/s12913-020-05159-9)
Supplement: Supplementary file 2 — Additional file 2. Initial ‘If… then’ statements (pre-testing): ‘If…then’ statements, developed from the literature, The Best Start plan and stakeholder interviews. [file 12913_2020_5159_MOESM2_ESM.docx]

**Additional File 1. Initial ‘If… then’ statements (pre-testing)**

1. If women and midwives build trusting relationships then women feel known and understood and will feel confident that their midwife will provide good care that is acceptable to them. They will trust in their midwife which makes them feel relaxed, less anxious and motivated to engage with health advice
2. If women feel accepted for who they are rather than feeling watched or judged they will feel relaxed and be more likely to disclose personal information and seek out more information. This gives the midwife and the woman more information to inform decision making and plan individualised care
3. If women feel valued and cared they will engage with services and feel more confident in themselves and their own abilities to grow, birth and nurture their baby.
4. If midwives get to know their women over a period of time they will plan individualised and appropriate care and will detect changes to health or emotional wellbeing, ensuring timely identification, treatment or referral
5. If midwives know their women they will find caring for them during labour and birth to be less stressful because they understand each other and have developed good communication
6. If midwives feel responsible for their women, they will want her to have the best experience and outcomes and will be motivated to ensure care is of the highest quality, evidence based and individualised.
7. If midwives and the MDT share the same philosophies and values then they will communicate openly about work and personal challenges allowing them to develop acceptable solutions for problems
8. If midwives feel accepted for who they are rather than feeling watched or judged they will feel relaxed and less anxious about how they are providing care and will be able to ask for (and provide) support and help from each other and the wider MDT, particularly in unfamiliar or challenging situations
9. If midwives value and care for each other then they will work together to ensure a good work life balance and professional experience and will feel sustained
10. If midwives feel valued and cared for within the wider context they will feel supported and empowered to provide high quality care and to engage with and support the CMC model of care
11. If midwives feel known and understood then they will feel able to communicate openly about work and personal challenges allowing them to develop acceptable ways of enabling CMC to fit within the wider care context.
12. If midwives feel they are trusted by the organisation to be in control of their own work then they will be able to provide flexible woman-centred, high quality care and support the CMC model of care
13. If the organisation feels able to trust midwives to be in control of their diaries, work and be flexible in how they work then midwives will feel empowered to work autonomously.
14. If midwives believe in CMC they will be motivated to work to full scope of midwifery practice and will see the consequences of care decisions, motivating them to reflect on practice and seek out the best quality evidence to ensure women have good outcomes
15. If midwives believe in CMC they will be motivated to support implementation even if they choose not to be part of a CMC team. They will provide expert, informational and emotional support and direct resources to women, midwives and the MDT to enable and CMC and will speak out against negative attitudes or behaviours that don’t support women, midwives’ practice or CMC
16. If MWs feel responsible for all midwifery care across the care journey they share information effectively to support fully informed choices, involve women in holistic woman centred decisions and actively seek out best available evidence
17. If midwives provide care across the whole care journey rather than fragmented by different locations, they are no longer ‘in a bubble’ and can see how care decisions play out. By reflecting on care experiences and outcomes the midwives choose to change their practice to become more evidence based and women centred
18. If midwifes feel in control of their diary they will work autonomously and flexibly, will plan their work around the needs of women, colleagues and themselves and will develop different methods of communicating and keeping in touch which will make women feel safe and cared for
19. If midwives see women in their home environment they will get to know her as a person, will remember her (see relationships), get to know her needs, her family, and support networks and will have more information and understanding to tailor information to suit her needs (see relationships)
20. If midwives and wider workforce see and feel supported by good leadership aligned with the values and principles of CMC and their concerns are heard and addressed they will feel reassured, will trust the process will be well managed, their jobs will be secure and that ‘someone has their back’ motivating them to support implementation of CMC.
