# Supplementary material for: Implementing continuity of midwife carer – just a friendly face? A realist evaluation
Source: BMC Health Serv Res. 2020 Apr 15;20:304. doi: 10.1186/s12913-020-05159-9 (PMC7158105; doi:10.1186/s12913-020-05159-9)
Supplement: Supplementary file 3 — Additional file 3. Preliminary CMO configurations: these were developed from the ‘If…then’ statements and tested in the on-going evaluation. [file 12913_2020_5159_MOESM3_ESM.docx]

**Additional File 2 – Preliminary CMO configurations**

| **Relationships** |  |  |
| --- | --- | --- |
| *Context* | *Mechanisms* | *Outcomes* |
| Positive relationships between midwives and women, between midwives regardless of where they work and between MW & the MDT | - Women get to know their midwife, which builds trust, this makes them feel safe, relaxed, empowered and confident. Midwives get to know women, which builds trust and confidence and sense of responsibility. - Midwives are empowered because they trust in each other’s skills, share responsibility and provide positive support - Midwives and managers are empowered because they feel trusted and able to trust | Implementation of CMC model of care  Improved care giving experience, care culture, satisfaction with care and clinical outcomes  Better wellbeing |
| **Practice** |  |  |
| *Context* | *Mechanisms* | *Outcomes* |
| CMC enables midwives to practise to full skill set | - Empowered to practice autonomously, provide flexible, holistic care, use an evidence informed approach to meet women’s individual needs, ensure women are at the centre of their care | Implementation of CMC model of care  Woman centred (individualised) care and satisfaction with care  Evidence based care  Confidence  Development and Midwife role satisfaction |
| Midwives believe in CMC/ have positive experience of CMC / already practising, or trying to practise, in a holistic woman centred manner. | - Motivated to engage and support CMC. |  |
| **Leadership** |  |  |
| *Context* | *Mechanisms* | *Outcomes* |
| Good leadership and management of organisational change | Mutual trust and feeling supported  Enable to engage with change required to make CMC happen | CMC model implemented  Midwives feel in control of their work and have a good work-life balance |
