# Supplementary material for: Implementing continuity of midwife carer – just a friendly face? A realist evaluation
Source: BMC Health Serv Res. 2020 Apr 15;20:304. doi: 10.1186/s12913-020-05159-9 (PMC7158105; doi:10.1186/s12913-020-05159-9)
Supplement: Supplementary file 4 — Additional file 4 Additional ‘If ……then’ statements from testing. [file 12913_2020_5159_MOESM4_ESM.docx]

**Additional File 3:** Additional **'**If ……then’ statements from testing

| 1. If the midwife does not know the women she is caring for during labour & birth (as per current teams model) AND is providing care within an unsupportive hospital /fragmented model context then she will feel more anxious and stressed and less in control of her ability to provide high quality care. |
| --- |
| 1. If midwives feel responsible for their women and want her to have the best experience and outcomes BUT are not supported by the prevailing system and care practices then they will not be able to meet their woman’s needs or their own expectations. |
| 1. If midwives feel responsible for their women and want her to have the best experience and outcomes BUT are not supported by the prevailing system and care practices AND feel undermined by poor relationships with wider workforce then they will not be able to meet their woman’s needs or their own expectations. |
| 1. If midwives feel responsible for their women BUT are not supported by the prevailing system and care practices to be present at all care appointment including labour and birth then they might feel a sense of abandonment. |
| 1. Likewise if women are not attended at birth by their midwife they will feel let down and disappointed. |
| 1. If team/all midwives feel watched and judged by the wider workforce then they will feel defensive, undermined and unable to seek help or support in challenging situation and/or unfamiliar environments. |
| 1. If team midwives do not feel valued or cared for by the wider workforce then they will not feel they belong, will feel defensive and demotivated to be present in this environment. |
| 1. If there is a lack of shared understanding between team and wider midwives then communication will not be open or honest and both groups will struggle to accommodate each other’s needs or the needs of the different ways of working. |
| 1. If team midwives do not feel valued and cared for then they will feel unsupported which in the current fragmented model context leaves them feeling exposed and vulnerable. |
| 1. If team midwives do not feel valued and cared for then they will feel unsupported, exposed and vulnerable. This will feel overwhelming and disempowering within the context of the prevailing fragmented model. |
| 1. If team midwives do not feel trusted or are micromanaged or judged by the organisation they will not feel in control of their work and will not be able to work flexibly or woman-centred. |
| 1. If midwives do not see trust and support or that team midwives are valued and cared for by the organisation then they will fear how the CMC will affect them, will not feel motivated to support CMC or the midwives and may actively or covertly work to ensure it does not happen. |
| 1. If midwives do not trust the organisation to manage the implementation of CMC properly or have had previous poor experience of change management within the organisation than they will not engage with the changes required because they are frightened of the impact on them personally and professionally. |
| 1. If the organisation feels unable to trust the CMC midwives or relinquish control of how they manage their workload and working life then they will judge, interfere with or micromanage processes which will disempower CMC midwives and prevent autonomous practice. |
| 1. If the organisation values the status quo, current systems and wants to retain an organisation focused rather than women centred approach to care then they will not feel able to actively support the implementation of CMC and will not build positive supportive relationships with CMC midwives. |
| 1. If midwives or wider workforce don’t believe in or value CMC they will not feel motivated to engage with the changes required to implement and sustain CMC, some may be concerned that outcomes or care experiences will be poorer in this model reinforcing their lack of involvement. |
| 1. If midwives feel they are already proving high quality continuity of carer and feel proud of their care then they will feel angry and undervalued because they feel they and their care have been judged and found to be wanting, this means they will not engage with the changes required and may covertly or overtly undermine efforts in order to preserve the good quality care they already have. |
| 1. If midwives believe that CMC means they will be unable to sustain a happy, health family and social life they will actively undermine CMC to preserve their current work-life balance because they feel they are losing control of their own lives and frightened about the effects on them. |
| 1. If MWs do not have experience of flexible working or managing a caseload and have not had the opportunity to build or develop these skills then this will feel daunting and insurmountable and may cause additional stress for team midwives or may deter others from taking part. |
| 1. If midwives do not feel in control or able to be fully autonomous then they will not be able to provide woman centred care and will feel demotivated to continue engaging with CMC. |
| 1. If midwives do not feel supported to provide care flexibly or are forced to use organisational facilities, they will be unable to provide high quality woman centred care and will not feel able to practice autonomously. |
| 1. If practices at the meso level (wider workforce) and organisational level (macro) remain unchanged it will be hard to accommodate CMC because the current systems are designed for fragmented care and do not support autonomous or flexible practice. |
| 1. If there is no shared vision or goals between those in CMC and those in FM expectations around staffing will be unmet and some will feel that team midwives are getting more favourable treatment leading to resentment and a breakdown in working relationships. |
| 1. If midwives and the wider workforce do not see or experience good leadership they will feel unsupported and will fear the consequences of change, this means they will not engage with the change process, may overtly or covertly undermine efforts to make CMC happen or may leave. |
| 1. If the organisation is unable to, does not want to, or is frightened to, change or release control of working practices to enable midwives to work autonomously and flexibly to provide high quality evidence based care then they will overtly or covertly undermine efforts to make CMC happen. |
| 1. If they do not trust midwives to work autonomously and flexibly to provide high quality evidence based care then they will watch, judge and micromanage midwives roles, midwives will feel undermined and resentful and will disengage from CMC. |
